# Retraction: Application of Intelligent Computer-Assisted Taylor 3D External Fixation in the Treatment of Tibiofibular Fracture: Retrospective Case Study

**DOI:** 10.2196/98674

**Published:** 2026-04-28

**Authors:** JMIR Publications Editorial Office

**Affiliations:** 1 JMIR Publications Toronto, Ontario

The JMIR Publications Editorial Office is retracting the article:

Application of Intelligent Computer-Assisted Taylor 3D External Fixation in the Treatment of Tibiofibular Fracture: Retrospective Case Study [1]

This action is being taken due to identified concerns regarding potential manipulation of the submission process and authorship, the integrity of the peer review process, and the source of the study’s ethics approval.

The concerns with this publication were identified during an investigation of a series of articles with related concerns. The authors were contacted and offered an opportunity to comment on these findings and the proposed retraction but did not respond to any attempts at communication.
